# Supplementary material for: HIGH-DIMENSIONAL MULTI-OMIC MAPPING OF POST-MORTEM HUMAN BRAIN USING ITERATIVE INDIRECT IMMUNOFLUORESCENCE IMAGING ON XENIUM-PROCESSED TISSUES
Source: bioRxiv. 2026 Sep 23:2026.09.16.751033. Preprint. [Version 1] doi: 10.64898/2026.09.16.751033 (PMC13622011; doi:10.64898/2026.09.16.751033)
Supplement: Supplementary file 1 [file media-1.pdf]

Supplementary Figure 1

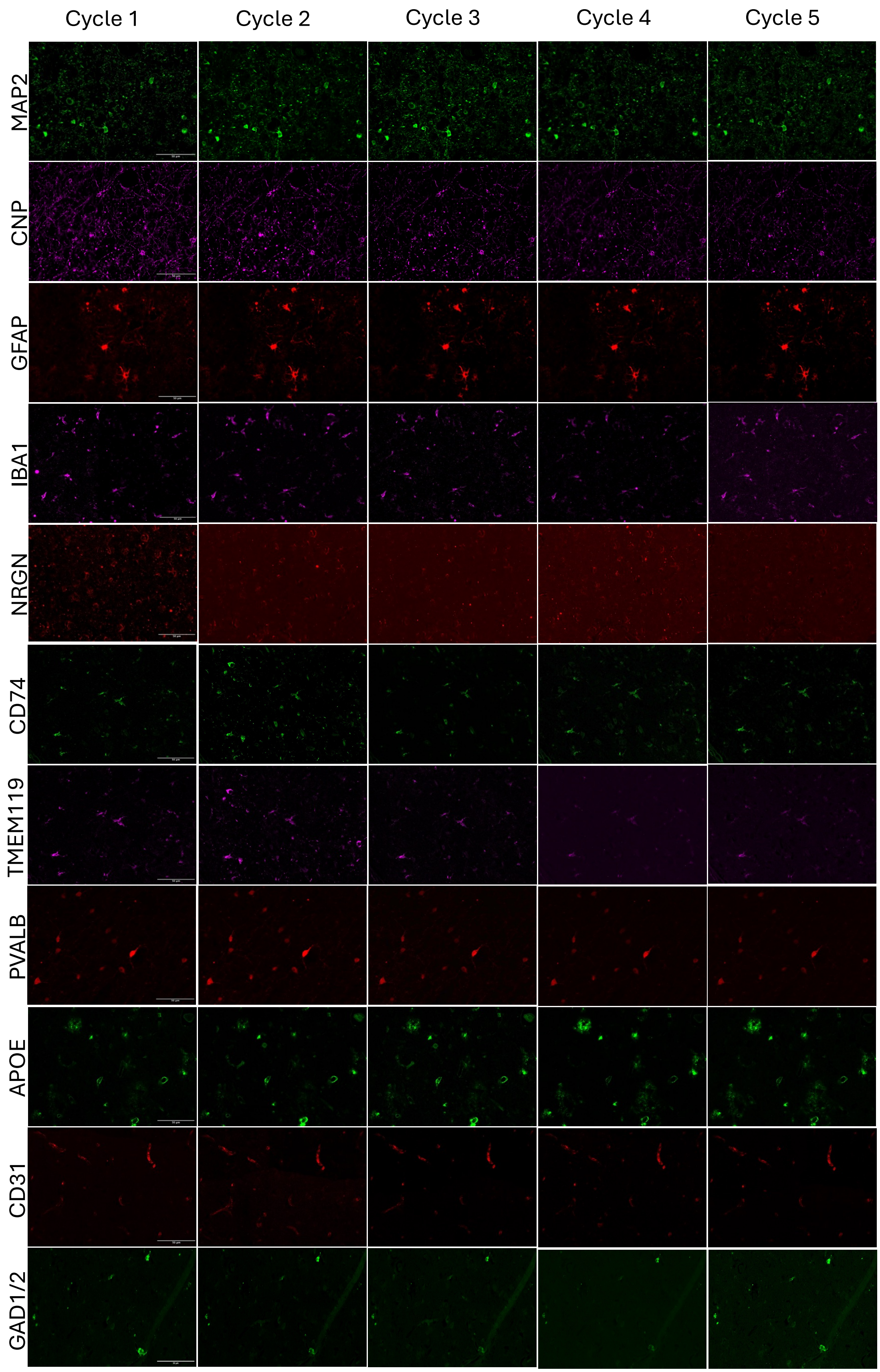

Supplementary Figure 2

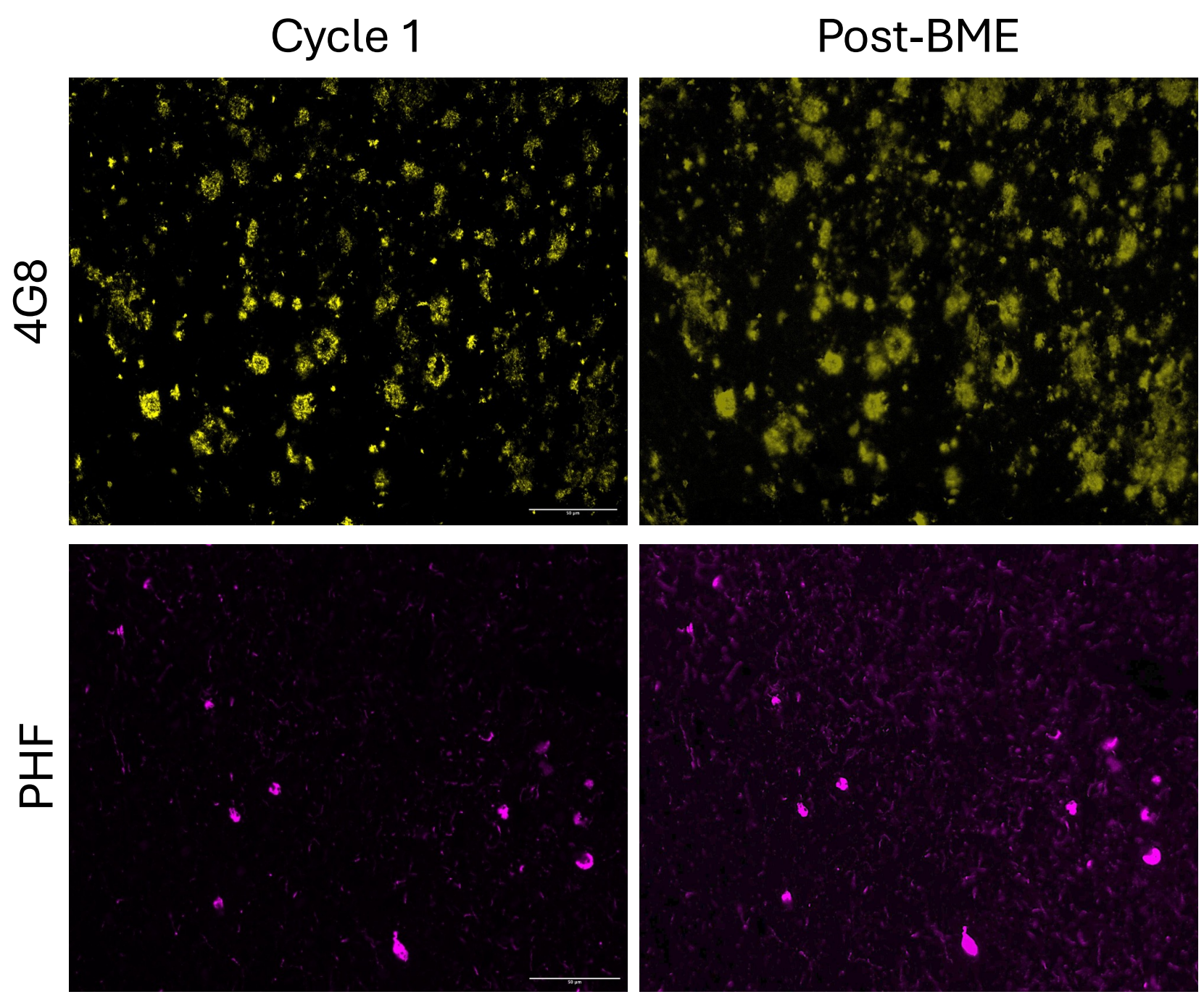

Supplementary Figure 3

a β-mercaptoethanol Stripping Solution

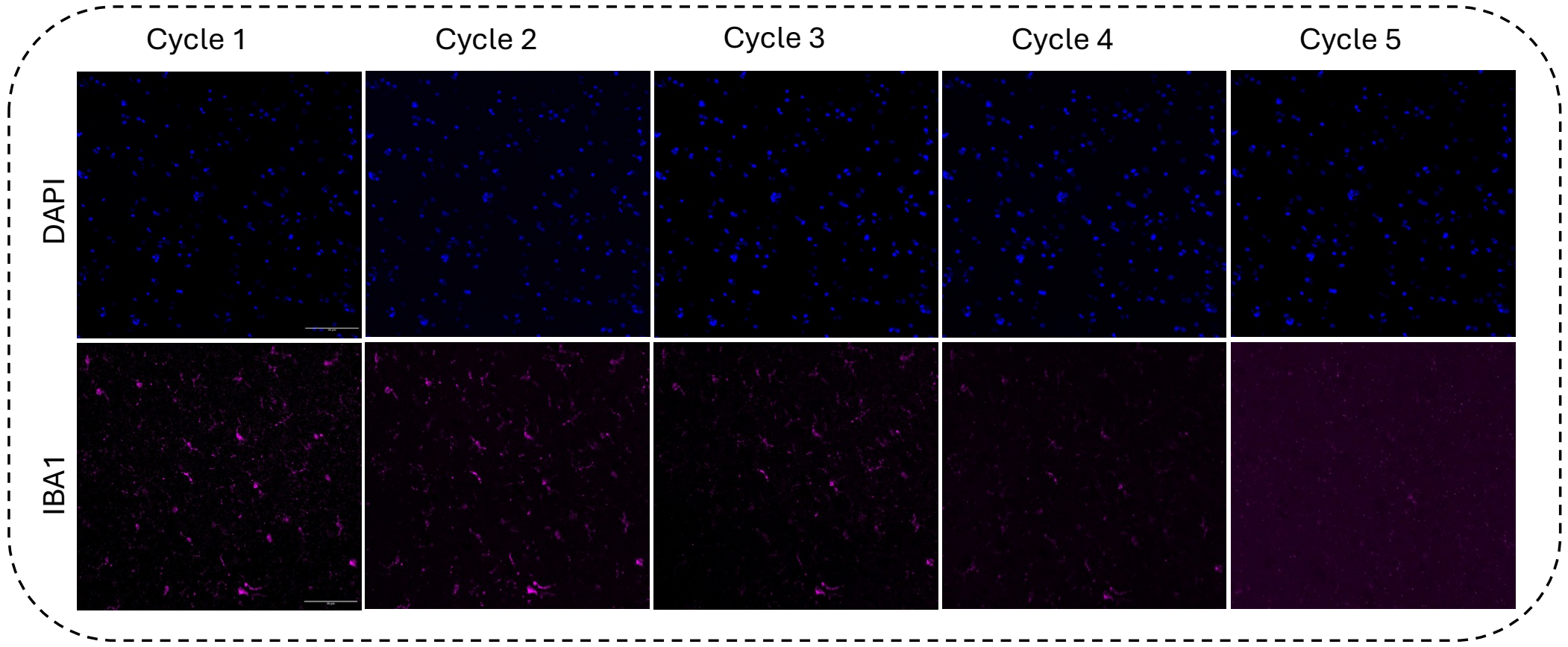

b Glycine Stripping Solution

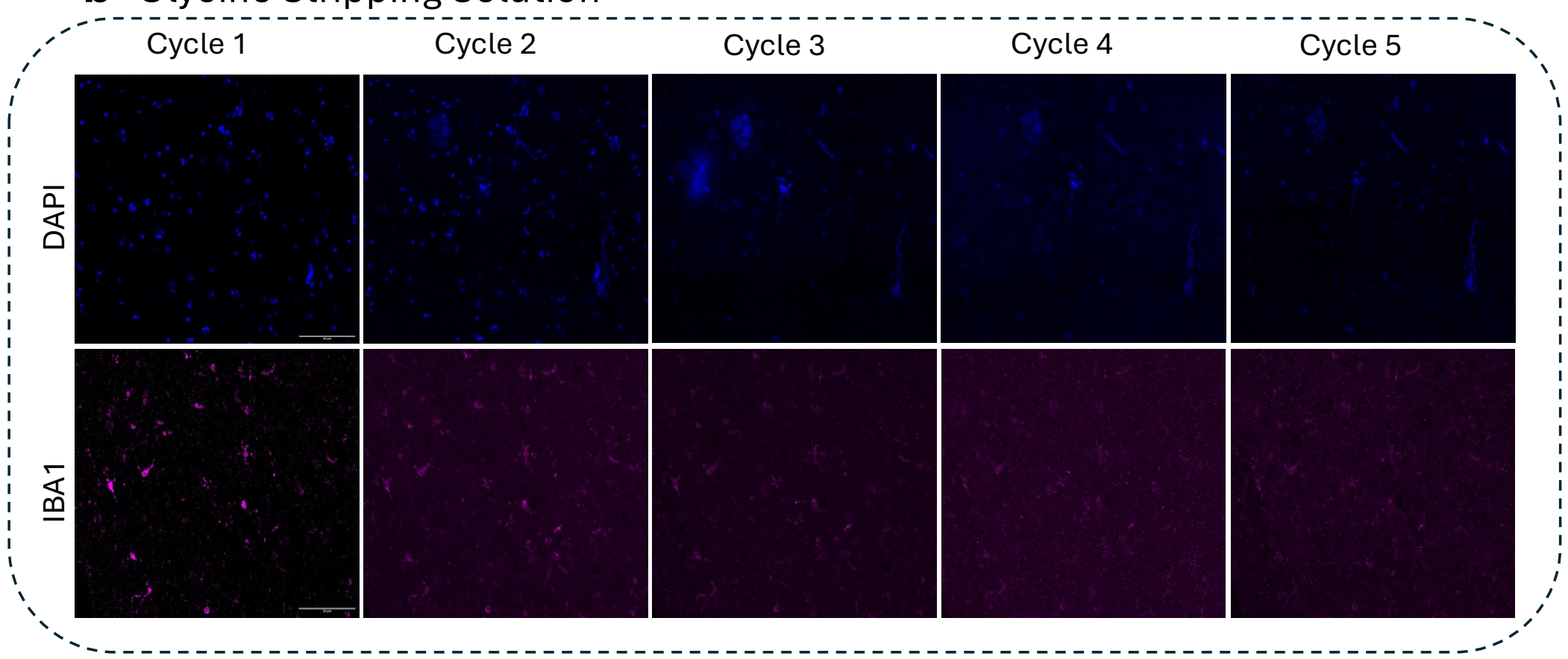

Supplementary Figure 4

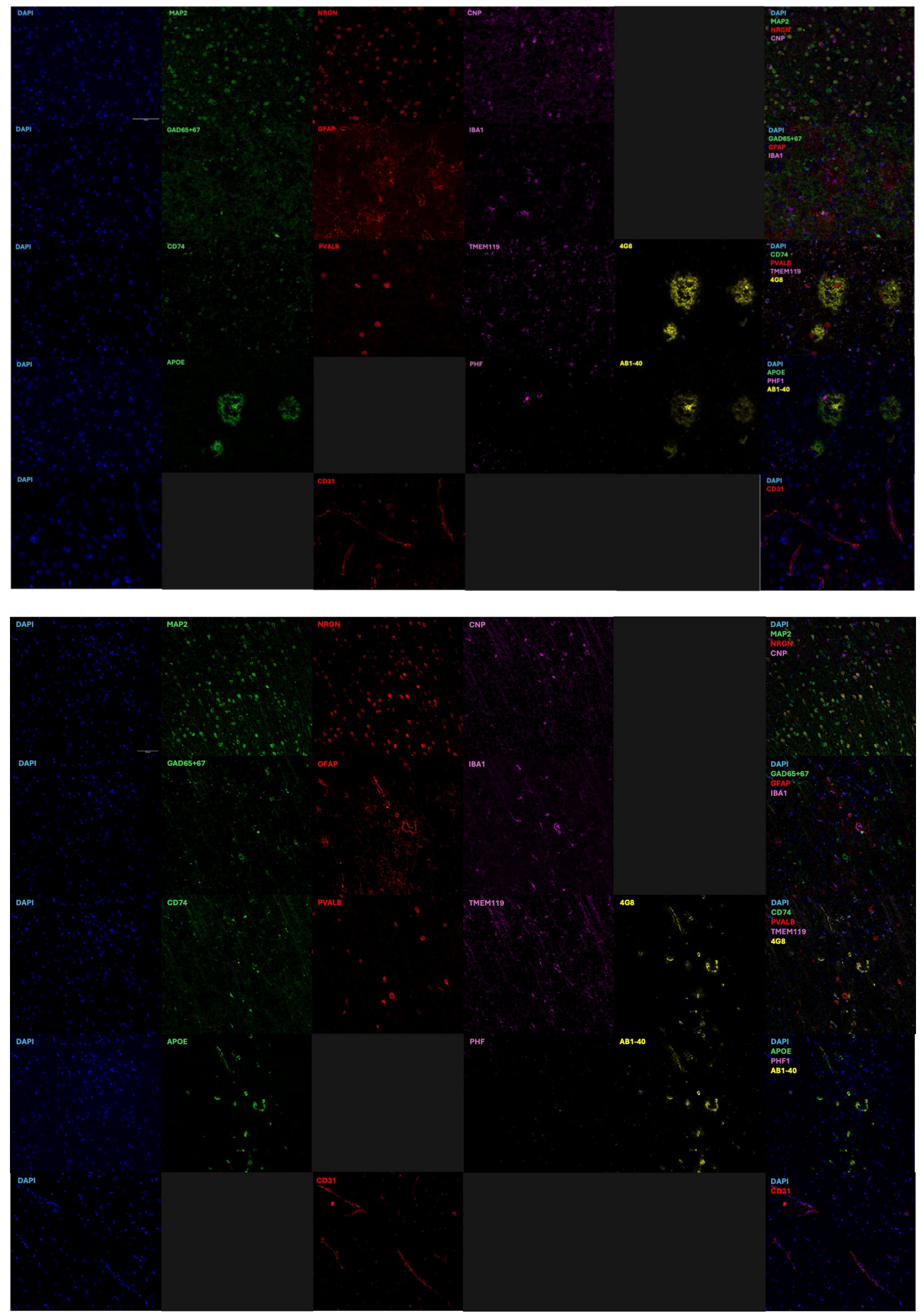

Supplementary Figure 5

a

	Marker	Training n = 11	Validation n = 2	Testing n = 2
Neurons	MAP2	35652	6682	6352
Microglia	Iba1	6840	1255	1039
Astrocytes	GFAP	5447	633	1331
Oligodendrocytes	CNP	5493	1157	1062
Vasculature	Morphology	4041	888	501
Unknown	N/A	1626	294	207
**Total**		**59,099**	**10,909**	**10,492**

b

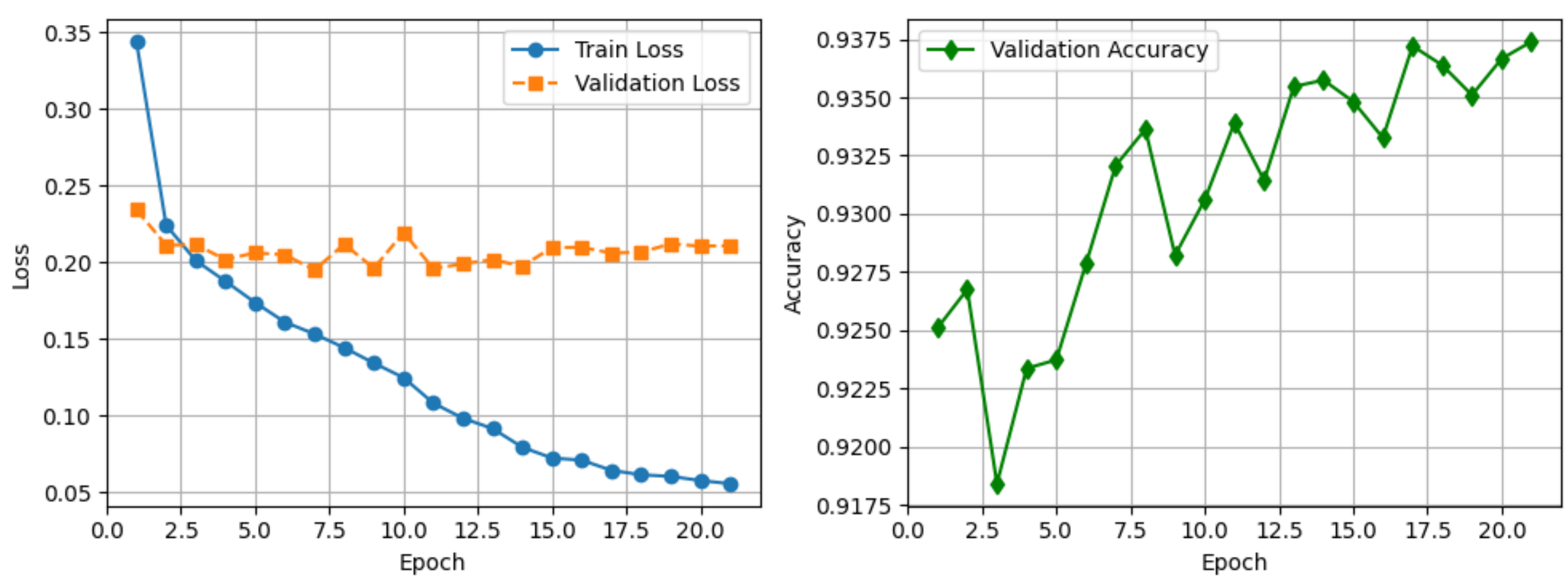

Supplementary Figure 6

a

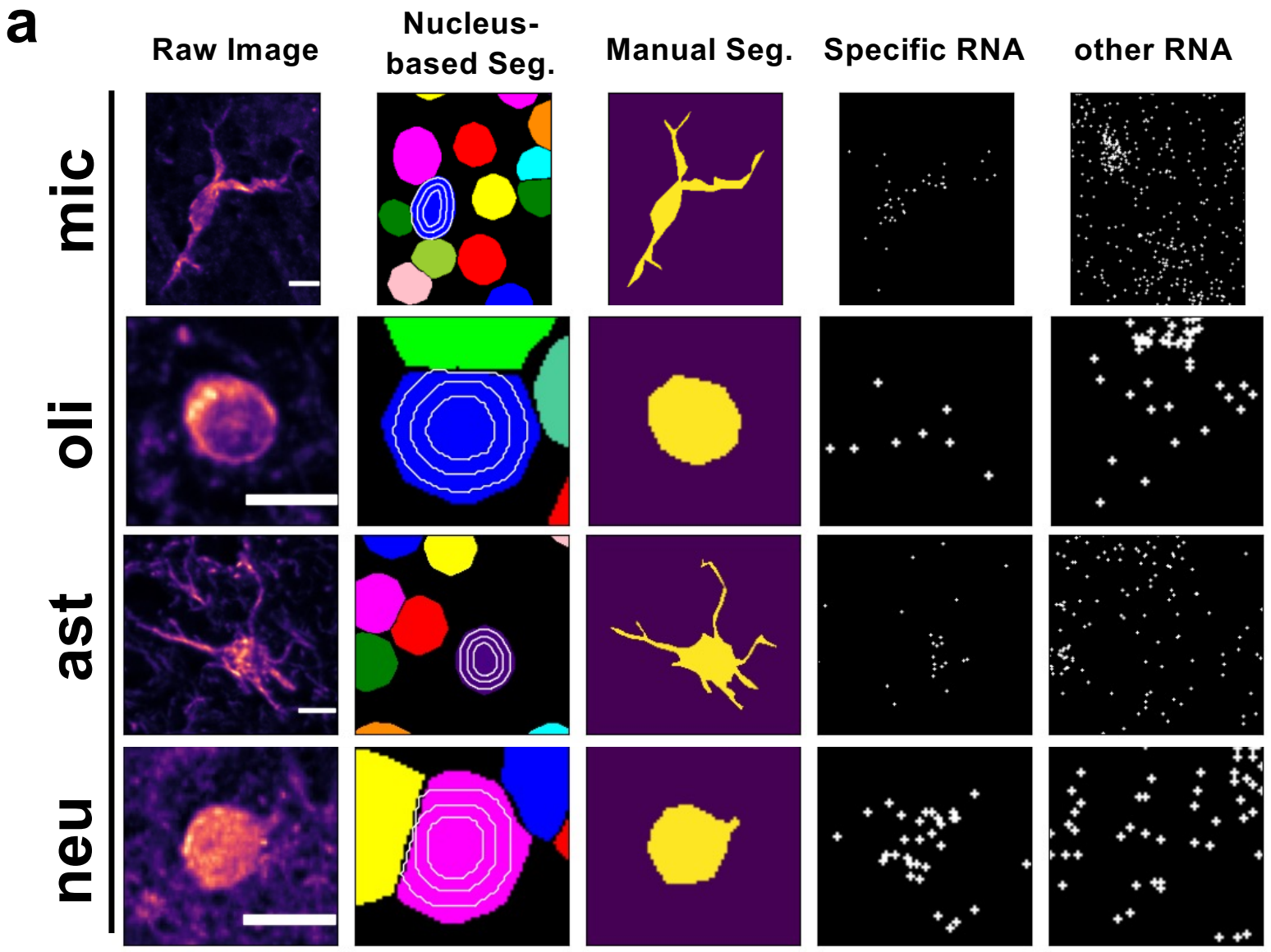

b

	Manual Seg.	Nucleus Only	5µm Expansion	50% Expansion	100% Expansion
**f1**					
mic	0.525	0.304	0.535	0.459	0.508
oli	0.473	0.450	0.423	0.454	0.436
ast	0.304	0.142	0.327	0.241	0.300
neu	0.686	0.386	0.769	0.684	0.763
**nCount**					
mic	16.675	6.525	18.877	12.581	16.315
oli	5.199	2.652	8.402	5.086	6.741
ast	14.180	4.482	14.041	8.696	12.821
neu	28.694	12.669	36.238	27.786	35.181

Supplementary Table 1

projid	ID	Age of Death	Sex	NIA-Reagan	Cognition	CAA	APOE genotype
02899847	xenium_1	74	male	Low	MCI	Moderate	33
03713990	xenium_2	88	male	Int.	NCI	Mild	33
10101741	xenium_3	90+	male	High	AD	Mild	33
10248033	xenium_4	88	male	Int.	AD	Moderate	44
10425642	xenium_5	87	male	High	AD	Mild	33
17929065	xenium_6	90+	female	Low	NCI	Mild	33
18414513	xenium_7	90+	male	Int.	AD	Moderate	33
20372539	xenium_8	86	female	High	AD	Severe	44
33321607	xenium_9	90+	male	Low	NCI	Mild	33
50302392	xenium_10	90+	female	High	AD	Mild	33
50401002	xenium_11	86	female	Int.	NCI	Mild	33
54122640	xenium_12	90+	female	High	AD	Moderate	33
59150662	xenium_13	90+	female	Int.	AD	Mild	33
77180612	xenium_14	87	female	High	AD	Severe	33
94328246	xenium_15	90+	male	High	MCI	Moderate	33

Supplementary Table 2

Cycle	Fluorophore			
	**Alexa Fluor 488**	**Alexa Fluor 568**	**Alexa Fluor 647**	**Alexa Fluor 790**
**1**	MAP2	NRGN	CNP	
**2**	GAD65+GAD67	GFAP	IBA1	
**3**	CD74	PVALB	TMEM119	β-Amyloid, 17-24
**4**	APOE		Tau (phospho S396)	β-Amyloid, 1-40
**5**		CD31		

Supplementary Table 3

REAGENT or RESOURCE	SOURCE	IDENTIFIER
Antibodies		
Chicken polyclonal anti-MAP2 (1:500)	Novus Biologicals	Cat#NB300-213; RRID: AB_2138178
Mouse monoclonal anti-NRGN [C2] (1:100)	Invitrogen	Cat#MA5-41648; RRID: AB_2899130
Rabbit polyclonal anti-CNP (1:100)	Sigma-Aldrich	Cat#HPA023278; RRID: AB_1847060
Rabbit monoclonal anti-GAD65+GAD67 [EPR19366] (1:100)	Abcam	Cat#ab183999; RRID: AB_3662875
Mouse monoclonal anti-GFAP, Cy3 conjugate (1:100)	Sigma-Aldrich	Cat#MAB3402C3; RRID: AB_11213580
Goat polyclonal anti-IBA1 (1:100)	FUJIFILM Wako Pure Chemical Corporation	Cat#011-27991; RRID: AB_2935833
Rabbit polyclonal anti-CD74 (1:100)	Sigma-Aldrich	Cat#HPA010592; RRID: AB_1078482
Sheep polyclonal anti-PVALB (1:300)	Invitrogen	Cat#PA5-47693; RRID: AB_2609239
Rabbit monoclonal Alexa Fluor® 647 anti-TMEM119 [EPR25865-89] (1:100)	Abcam	Cat#ab313674
Mouse monoclonal anti-β-Amyloid, 17-24 (1:16000)	BioLegend	Cat#800702; RRID: AB_2564634
Goat polyclonal anti-APOE (1:300)	Invitrogen	Cat#PA5-18361; RRID: AB_10979861
Rabbit monoclonal anti-Tau (phospho S396) [EPR2731] (1:100)	Abcam	Cat#ab156623
Mouse monoclonal anti-β-Amyloid, 1-40 (1:300)	BioLegend	Cat#805402; RRID: AB_2564681
Rabbit polyclonal anti-CD31 (1:50)	Abcam	Cat#ab28364; RRID: AB_726362
Donkey anti-Rabbit IgG (H+L) Highly Cross-Adsorbed Secondary Antibody, Alexa Fluor™ 488 (1:500)	Invitrogen	Cat#A-21206; RRID: AB_2535792
Donkey anti-Goat IgG (H+L) Cross-Adsorbed Secondary Antibody, Alexa Fluor™ 488 (1:500)	Invitrogen	Cat#A-11055; RRID: AB_2534102
Donkey anti-Chicken IgY (H+L) Highly Cross Adsorbed Secondary Antibody, Alexa Fluor™ 488 (1:500)	Invitrogen	Cat#A78948; RRID: AB_2921070
Donkey anti-Mouse IgG (H+L) Highly Cross-Adsorbed Secondary Antibody, Alexa Fluor™ 568 (1:500)	Invitrogen	Cat#A10037; RRID: AB_11180865
Donkey anti-Rabbit IgG (H+L) Highly Cross-Adsorbed Secondary Antibody, Alexa Fluor™ 568 (1:500)	Invitrogen	Cat#A10042; RRID: AB_2534017
Donkey anti-Sheep IgG (H+L) Cross-Adsorbed Secondary Antibody, Alexa Fluor™ 568 (1:500)	Invitrogen	Cat#A-21099; RRID: AB_2535753
Donkey anti-Rabbit IgG (H+L) Highly Cross-Adsorbed Secondary Antibody, Alexa Fluor™ 647 (1:500)	Invitrogen	Cat#A-31573; RRID: AB_2536183
